# Diagnostic Value of the Soft Palate, as Compared with the Tongue in Certain Pathological Conditions

**Published:** 1885-02

**Authors:** Wm. Abram Love

**Affiliations:** Professor of Physiology in the Atlanta Medical College, Atlanta, Georgia


THE ATLANTA

MEDICAL AND SURGICAL JOURNAL.

VOL. I. FEBRUARY, 1885. No. 12.

### Original Communications.

### DIAGNOSTIC VALUE OF THE SOFT PALATE, AS COMPARED WITH THE TONGUE, IN CERTAIN PATHOLOGICAL CONDITIONS.*

BY WM. ABRAM LOVE, M D.

PROFESSOR OF PHYSIOLOGY IN THE ATLANTA MEDICAL COLLEGE, ATLANTA, GEORGIA.

In the current medical literature of the day—in the authorized text-books of our schools, and, more particularly, in the standard works of the older writers, much stress is placed upon the appearance of the *tongue*, as an index to certain pathological conditions of the system. In an especial sense were these varied appearances regarded as indicative of the condition of the stomach, the intestinal canal, and the hepatic secretions. That these appearances were sometimes erroneously interpreted, was very vividly impressed upon my mind, in my early professional life, by a foot-note in a small but valuable work then recently from the press. I may be excused for quoting that foot-note in its entirety, in this connection, as it will serve me a purpose, just here, and may, as it has me, serve others a purpose elsewhere.

*This paper appeared in the Transactions of the Medical Association of Georgia several years ago. On account of its value the author has rewritten it for THE JOURNAL. Our readers will find much new matter in this that was not in the original paper.—EDS.

In describing, for illustration, a case of "clavus hystericus of the head, kept up by inanition," the writer says [Billing's Principles of Medicine—Amer. Edit., 1842—p. 220-1.]: "——there was no fever; the pulse was jerking, as we find after hemorrhage (repeated blood-letting), but not firm; the tongue not foul, but white, as we always find it with an empty stomach." Then comes the footnote:

"I say, always; and there is not a more common error, than to consider this natural appearance morbid. Thus, persons who are in the habit of thinking themselves bilious, and taking physic, look at their tongue when they rise in the morning, and find it white. A good breakfast will make it look red, unless they take a dose of salts, Seidlitz powder, or sometimes even whether they do or not. The same person will, perhaps, put out the tongue before a looking-glass just before dinner time, and, seeing it white, forego a part of a wholesome meal, which would bring the tongue to the natural color of redness which it assumes after eating, from its natural paleness before eating, unless they be gourmands and hypochondriacs at the same time; in which case they will run the hazard of eating, and take a calomel 'peristaltic persuader' afterwards. I have been, constantly, in the habit of warning my young medical friends to consider, when they see a white tongue, what time of day it is, and *not to purge* for merely a white, or, more properly, a pale tongue.

"The tongue is constantly very properly inspected, in disease, as it affords an evidence of the state of the mucous membrane of the stomach and bowels with which it is continuous. In health it is not of a bright red, but has a pale bloom on its surface, in consequence of the tips of the villi or papillæ being less injected with blood than the lower parts; when the stomach is empty, it contains less blood, its villi are, of course, paler, and those of the tongue are nearly white; but observe, the tongue is moist; whereas, in the beginning of synocha or pleurisy, or other inflammation, the stomach is empty from anorexia, and the tongue is white; but it becomes drier than from a mere empty stomach and more or less coated, arising from the evaporation of the watery parts of the saliva and mucus of the mouth, which leaves the membrane indued with a more viscid covering than natural. After eating, when the stomach is in a state of healthy activity, the tongue becomes redder; but still it is not of a bright red hue, which only takes place when the membrane of the primæ viæ is in a congested or inflamed state, as in dysentery, in phthisis when colliquative diarrhœa exists. at the termination of typhoid fever when there has been (in reality) gastro-enteritis or inflammation of the glandulæ agminatæ, etc.

"In the progress of severe fever, when the secretions are suspended, the tongue becomes dry, and the mucus which does exist dries, and forms a brownish or blackish crust, and the papillæ become so much shrunk down to the level of the rete mucosum, that when the tongue becomes clean, on recovery, it looks glazed and smooth, and some time elapses before the papillæ rise up again.

"In chronic affections, accompanied with a languid and flabby state of the primæ viæ, a discolored state of the mucus occurs, constituting what is called a foul tongue."

Few more concise descriptions of the pathognomonic indications presented by the appearance of the tongue, will be found in our medical literature; but these relate more particularly to the appearance of the upper surface of the tongue as indicating *physiological,* or *pathological* conditions of the mucous membrane of the stomach and intestinal tube, yet the tongue is in many cases—as well as other portions of the oral cavity—an indicator, pointing to many and varied pathological conditions of the general system, and of special tissues. As a contribution illustrative of this may be mentioned "*a peculiar appearance of the tongue in malarious diseases.*"

While the appearance of the tongue indicative of physiological and pathological conditions of the alimentary mucous membrane presents itself on the upper papillated surface—the *border* and *outer edges* present the peculiarity indicative of malarial toxæmia. It consists in a peculiar *pectiniform* appearance of the edges of the tongue, as though these edges had been under the pressure of the sides of the teeth of a comb—just as, in certain "languid and flabby" states of the primæ viæ we find the edges presenting a crenated appearance, produced by the indentations resulting from the pressure of the teeth in the oral cavity—just within this *pectiniform edge* making the outer border of the upper surface of greater or less width, in different cases, or different degrees of malarial toxæmia, there appears a *smooth margin*, both the *pectiniform edge* and the *smooth margin* presenting a *cleaner appearance* and *a brighter hue* than the other portions of the surface of the organ. For over thirty years, in an active practice, most of the time within malarial districts, these peculiarities of the edges and borders of the tongue have been marked as indicative of malarial poison, not only in the malarial fevers of the paludal districts, but in the protean forms and varied complications through which the effects of this subtle poison may be traced. While it had been my good fortune, in early professional life, to detect this condition as a pathognomonic indication of malarial poison, which experience, until this day, has more fully confirmed, and, while I have profited by it during these long years of professional toil, still, to my friend, Dr. T. C. Osborne, of Greensboro, Alabama, is due the credit of having first called the attention of the profession to the fact through the recognized channel of communication —the medical press (*vide Trans. Amer. Med. Assoc.*, 1869), and I take pleasure in awarding him this credit, with the expression of regret that his paper did not fall into the hands or attract the attention of more of my professional brethren.

Dr. Osborne has preferred the term '*crenated edge*" in his description given in his paper. This, to our mind, without an illustration (which he presents), would more nearly convey the idea of the indentations produced by the teeth in the oral cavity in the flabby condition alluded to above, when the term "*crenated*" is applied to such a body or organ as the tongue; we have, therefore, held to our original descriptive term, *pectinated*, or *pectiniform*, with the explanation, illustrative, given above.

These allusions to certain conditions of the tongue, as indicative of certain pathological conditions of the primæ viæ, or of the general system, have been made more particularly as introductory to, if not illustrative of, certain other conditions often presented in another portion of the oral cavity, and their value, in a diagnostic, as well as a pathological and therapeutical point of view. We allude to—

### THE VALUE OF THE APPEARANCE OF THE PALATINE VAULT AND SOFT PALATE IN DIAGNOSIS.

On the sides of a median line drawn from the point of the alveoli separating the two central superior incisor teeth to the center of the base of the velum palati, there are two elliptical, or almond-shaped spaces, where the inferior surface of the palatine bones is covered with only periosteum and mucous membrane, constituting, we will say the *palatine vault or dome.* An extension of this membrane backwards, united with a like extension from the superior surface of the bone, or the floor of the nasal cavity, to the velum palati and anterior half arches, constitutes *the soft palate*—the palatine muscles to some extent taking the place of the palatine bones. This muco-periosteal and muco-muscular membrane is supplied with blood by the superior palatine and naso-palatine arteries, whose branches anastomose with each other, and with their congeners. Their capillaries, particularly in the *palatine vault*, though exceedingly numerous, are, at the same time, exceedingly small, so much so that they allow of the passage of blood corpuscles to only a very limited extent in their normal condition, as in the conjunctiva, sclerotica and other membranes and tissues that circulate alone, liquor sanguinis normally, and blood corpuscles pathologically. The great vascularity (capillary) of both the mucous and the periosteal membrane, together with the great transparency of the same, and the bony and muscular base, gives us an opportunity of noting conditions that are of vast importance, both pathologically and therapeutically. Among these may be enumerated:

1st. The color of the liquor sanguinis;

2d. The arteriolic tension, or atony, in resistance or non-resistance to the passage of blood corpuscles; or, in other words, by the inspection of these spaces we are enabled to approximate an estimate of the amount of coloring matter (biliverdine) tinging the non corpuscular blood tissue, in the first place, and secondly, we are enabled to approximate pretty correctly the "working" of the vaso-motor nerve system, particularly along the line of the alimentary canal. (This does not apply, we would parenthesize, in cases of local irritation in these palatine tissues, except so far as relates to these local membranes.)

Practically these facts are of much advantage, and, for over a third of a century, I have been in the habit of taking advantage of them as guides in the diagnosis and treatment of disease. During this period the rule has been, with me, to examine the roof of the mouth as regularly as I have occasion to examine the tongue. So constant has been this habit with me, that I have been frequently asked the question by medical students: "*Why do you always examine the throats of your patients in the clinics?*"

I have found that in that condition of the system to which the term "*bilious*" has been applied, this muco-periosteal membrane invariably presents the yellow hue of lighter or deeper shade, indicative of the existence of *biliverdine* in the liquor sanguinis. This yellow tinge or color will vary in different cases, or at different times in the same case, from the lightest canary to the deepest orange or saffron, and the depth of shade will indicate the amount of "*biliousness*," or the extent to which biliary coloring matter is retained in the blood tissue. As this tinge deepens, the skin becomes more and more sallow, approaching towards that appearance exhibited in mild cases of acute jaundice. In all cases, under any and all circumstances, where bile has been "re-absorbed," or where it has not been eliminated from the blood—in malarial toxæmia—in duodinitis—in biliary cystitis—and in every condition of the system where, by its existence in the liquor sanguinis, or where, as a result of such pathological condition the tissues become tinged, the color will present itself first and deepest in the muco-periosteal membrane in the mouth as designated above. The only condition obstructing its appearance will be where there exists engorgement, irritation or inflammation, distending the minute capillaries to such an extent as to admit of the blood corpuscles, when the redness of the tissue swallows up the fainter yellow hues. By examining this portion of the roof of the mouth we gain a better knowledge of the condition of the portal system and hepatic action than the tongue indicates as to the condition of the stomach, in the circulation in its mucous membrane or the action of the gastric glands.

In all that class of diseases in which the general condition of the system demands the use of remedies known as *cholagogues*, of whatever kind, and in all forms and complications, experience has taught me that I risk nothing in saying that the muco-periosteal membrane in the roof of the mouth will by its yellow tinge invariably indicate the necessity for their administration; *per contra*, I may say, with equal confidence, that the absence of this yellowness indicates, with equal certainty, that such a class of remedies have been sufficiently used or are not needed. For thirty years this has been my guide, and I do not feel to-day that I have ever been misled by it. Other members of the profession, whose attention I have called to the fact long years since, tell me that as a guide in their daily professional work, it has served the same good office. Attention to it will do away with much of the use of, or rather *abuse of calomel.*

In other pathological conditions than this, the appearance of the palatine surface will serve us a good purpose as a guide. Thus, for example: in all that class of diseases known as *exanthema majora*, the eruption makes its appearance in the roof of the mouth, from *twelve to twenty-four hours*, and in many instances longer, before it appears on the cutaneous surface. In *smallpox*, in *scarlet fever*, in *measles*—in all their grades—the eruption may be looked for, with confidence in this region long before it can be detected at any other point, and, as the eruption is often the last link in the chain of evidence necessary to decide a question of diagnosis, the knowledge of this fact will always equal the importance of the question at issue; it has, in some instances, served me a valuable purpose.

In *intestinal* irritation and inflammation, in the approach, progress and decline of enteritis and dysentery, the soft palate is a better indicator as to the condition of the intestinal mucous membrane than the tongue.

In the rise and progress of such cases there is vascular engorgement of the palatine mucous membrane, indicating a like or worse condition along the line of the intestinal canal—a little attention to which will familiarize the practitioner with the varied changes in the appearance of the one, as pointing to the pathological conditions existing in the other.

In cases where this *yellow tinge of the soft palate* presents itself, another and more general disturbance will be found in the systemic capillary circulation. If, under pressure, the blood corpuscles are forced out of the cutaneous capillaries on the back of the hand or the wrist, the subcutaneous tissue will present, as seen through the skin, more or less of a yellow tinge, such as is seen in *the soft palate*, or in a case of jaundice, varying in different cases as to depth of shade. Not only this, but *the blood will be found to return tardily to the capillaries*, showing a passive capillary circulation not consistent with healthful action—a torpidity interrupting both assimilation and elimination. This condition results ultimately in a pathological accumulation of the effete products of physiological combustion in the organism. *These in turn produce depressions.* Beginning in the peripheral nerve filaments and ultimately affecting the nerve centres—the entire system becomes involved. The relation existing between the peripheral nerves and the capillary system and their reciprocal action, the one or the other, has not as yet been satisfactorily explained, still it is evident that the *non-elimination* of the effete products of combustion in the organism result in *depressions*—depression in the ganglionic system interfering with trophic action—with assimilation and disintegration—depression in the sensory system, giving rise to neuralgias and other disturbances of sensation—depression in the motor system, impairing action and tonicity of the skeletal muscles—depression in the cardio-motor and vaso-motor system, interfering with blood speed and blood pressure—all these disturbing healthful functional action. In these the effect adds continuously to the cause, the case becomes more and more complicated, until the tissues give way and organic disease is established where mere functional derangement had formerly existed.

These may seem trivial points in the investigation, but by giving them attention in time, by recognizing these facts and resorting to such remedies as will remove these dead elements—the smoke and ashes of physiological, perchance pathological—combustion that act like Dead Sea waters, supporting no living thing—serious consequences may be averted and healthful action restored.

